# Pre-stenting lesion preparation using shockwave intravascular lithotripsy in severely calcified superior mesenteric artery stenosis

**DOI:** 10.1186/s42155-025-00622-2

**Published:** 2026-01-07

**Authors:** Robert Terzis, Robert Wawer Matos Reimer, David Maintz, Erkan Celik

**Affiliations:** Institute for Diagnostic and Interventional Radiology, Faculty of Medicine and University Hospital Cologne, University of Cologne, Kerpener Straße 62, Cologne, 50937 Germany

## Abstract

**Background:**

Intravascular lithotripsy (IVL) is an emerging technique for modifying heavily calcified arterial lesions, with primary application in peripheral arteries. We report the use of IVL for lesion preparation prior to stenting in a patient with severely calcified superior mesenteric artery (SMA) stenosis.

**Case presentation:**

A 66-year-old man with type I adenocarcinoma of the esophagogastric junction (AEG Type I) and neoadjuvant FLOT chemotherapy was scheduled for Ivor Lewis esophagectomy. Preoperative CT angiography (CTA) revealed a high-grade ostial SMA stenosis due to extensive atherosclerotic calcification. To mitigate the risk of postoperative mesenteric hypoperfusion, percutaneous endovascular revascularization was performed. Following initial predilatation, IVL using a Shockwave 5.5 × 60 mm balloon catheter was employed for lesion preparation. Subsequently, an 8.0 × 24 mm balloon-expandable stent was successfully deployed with low-grade residual stenosis and no complications.

**Conclusion:**

This case demonstrates that IVL represents a feasible and effective adjunct in the management of severely calcified visceral arterial lesions. It facilitates adequate lesion preparation and enables full stent expansion, even when the IVL balloon diameter is notably smaller than the stent diameter. This potentially represents a less traumatic approach to the vessel than alternative techniques. IVL may therefore be considered a therapeutic option in selected patients.

## Background

Chronic mesenteric ischemia (CMI) commonly results from atherosclerotic narrowing of the mesenteric arteries and typically presents with postprandial pain and weight loss [1]. However, incidental SMA stenosis may also be detected during cancer staging or preoperative imaging. The SMA is the primary vessel supplying the small intestine. Its obstruction, especially in the context of limited collateral flow, can lead to clinically significant ischemia during physiologic stress, such as major abdominal surgery [2, 3].

Endovascular treatment options include percutaneous transluminal angioplasty (PTA) with stent placement [4]. However, dense calcification poses technical challenges and increases the risk of perforation, suboptimal expansion, recoil, or dissection [4]. Intravascular lithotripsy (IVL) has recently emerged as an innovative approach that facilitates calcium modification using sonic pressure waves to fracture intimal and medial calcifications, thus enhancing vessel compliance prior to stenting [5, 6].

## Case presentation

A 66-year-old male patient was diagnosed with adenocarcinoma of the esophagogastric junction type I (AEG Type I), with tumor extension from 40 to 44 cm from the alveolar ridge. He completed neoadjuvant chemotherapy per FLOT protocol. Follow-up CT staging revealed a regressive tumor with no distant metastases (cT2N0M0) but incidentally showed a high-grade ostial SMA stenosis of 91% due to heavily calcified atherosclerotic plaque, with an adjacent exophytic component extending into the aortic lumen. Given the impending Ivor Lewis esophagectomy and known hemodynamic dependence on SMA perfusion, interdisciplinary consensus recommended preoperative endovascular revascularization.

**Fig. 1:**
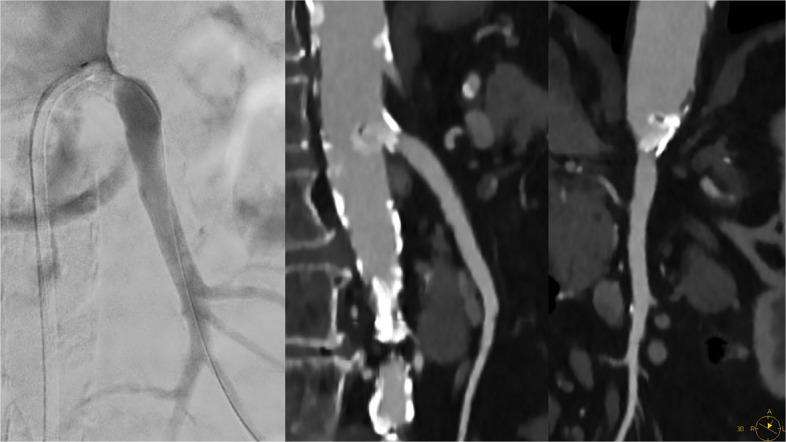
Selective angiography via femoral access confirmed a severe, 91% stenosis of the superior mesenteric artery. Pre-interventional CTA demonstrated a heavily calcified atherosclerotic plaque at the SMA origin, with an adjacent exophytic component extending into the abdominal aortic lumen

Under local anesthesia and continuous monitoring, percutaneous access was obtained via the right common femoral artery. A short 5 Fr sheath was introduced and exchanged for a 7 Fr steerable long sheath (Lamed GmbH, Oberhaching, Germany). A diagnostic angiogram confirmed a subtotal ostial SMA stenosis (Fig. 1). Intravascular imaging (e.g. intravascular ultrasound) was not performed to conserve procedural resources, given adequate angiographic visualization and the anticipated limited incremental yield.

**Fig. 2:**
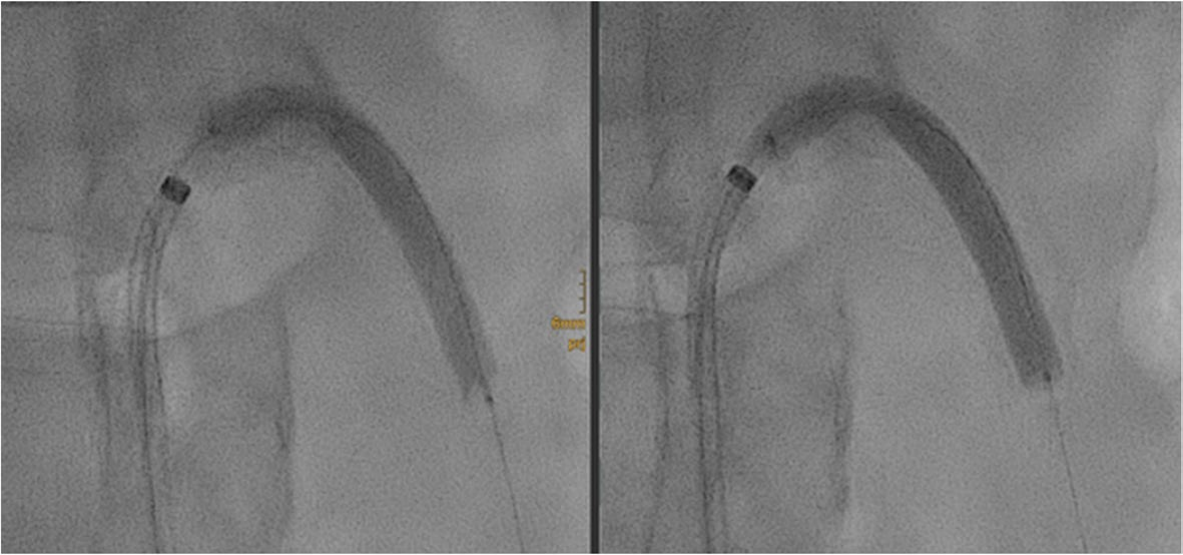
Initial (left) and final (right) cycles of intravascular lithotripsy using a 5.5 × 60 mm Shockwave balloon catheter, demonstrating complete coverage of the stenotic segment. Note how the application of sonic pressure waves progressively modifies the calcified plaque, facilitating gradual balloon expansion and improved vessel compliance over the course of treatment

After intraluminal recanalization, a 3.0 × 40 mm conventional balloon catheter (Sterling, Boston Scientific, Marlborough, MA, USA) was used for predilatation. Subsequently, a 5.5 × 60 mm Shockwave™ IVL balloon catheter (Shockwave Medical Inc., Santa Clara, CA, USA) was advanced into the SMA, and four full cycles of 30 electric pulses of lithotripsy were performed to treat the lesion (Fig. 2).

**Fig. 3:**
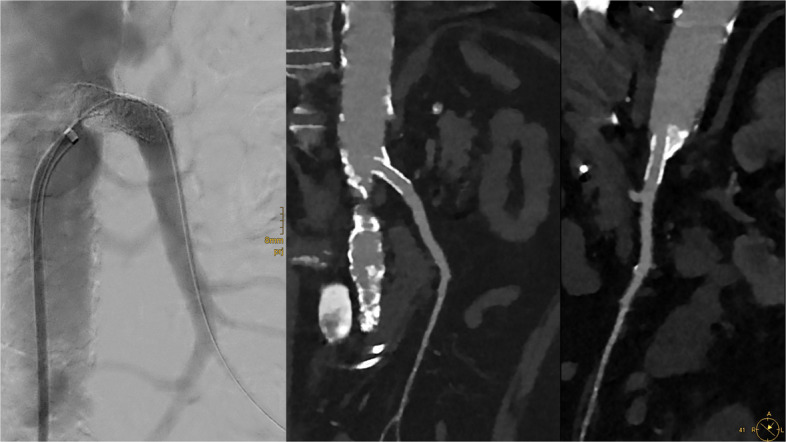
Post-treatment angiography following IVL and implantation of an 8.0 × 24 mm Formula balloon-expandable stent demonstrates successful revascularization of the SMA. Multiplanar reconstruction of the follow-up CTA after Ivor Lewis esophagectomy (14 days post stenting) confirms sustained vessel patency without evidence of restenosis or perioperative vascular compromise. Residual stenosis was 22%

An 8.0 × 24 mm Formula™ balloon-expandable stent (Cook Medical, Bloomington, IN, USA) was then implanted from the SMA ostium into the proximal main stem. Post-stenting angiography revealed a good angiographic result with a widely patent vessel and residual stenosis of only 22% (Fig. 3).

The arterial puncture site was closed by manual compression, followed by the application of a compression bandage. There were no peri- or postprocedural complications. The Ivor Lewis esophagectomy was successfully carried out as scheduled, without perioperative complications related to intestinal perfusion. At the 3-month postoperative follow-up, the patient was in good clinical condition with no adverse events, particularly no signs of mesenteric ischemia.

## Discussion

Endovascular revascularization has become the preferred treatment modality for chronic mesenteric ischemia due to its lower morbidity and mortality compared to open surgery, especially in high-risk surgical candidates or in the preoperative setting of major abdominal surgery [2–4]. However, severe ostial calcifications remain a major technical challenge, often impairing optimal stent deployment and increasing the risk of vessel perforation, elastic recoil, dissection, or restenosis [7]. IVL has recently emerged as a promising adjunctive technology to address these limitations. IVL uses pulsatile sonic pressure waves to fracture both intimal and medial calcium, thereby enhancing vessel compliance while minimizing trauma to the surrounding tissue [8]. Unlike atherectomy or high-pressure ballooning, IVL preserves the structural integrity of the vessel and does not generate particulate debris, reducing the risk of distal embolization [9, 10].

To date, most of the available data are derived from peripheral and coronary interventions, with large multicenter trials such as the DISRUPT PAD III and CAD III trials demonstrating the safety and efficacy of IVL in treating heavily calcified femoropopliteal and coronary lesions [10, 11]. In the latest report from the DISRUPT PAD III trial, IVL treatment of 164 common femoral artery lesions, 95.1% of which had moderate to severe calcification, resulted in a final residual stenosis of 23.6 ± 11.5% with no major vascular complications observed by core-laboratory assessment, underscoring its favorable safety profile even in anatomically complex and calcified vascular beds [5].

Specific evidence for IVL in the SMA is currently restricted to isolated case reports. Our findings are consistent with previous reports summarized in the most comprehensive review to date by Spath et al. [6], who compiled 11 cases involving IVL treatment of severely calcified visceral and renal arteries. The majority of cases (67%) addressed chronic mesenteric ischemia, with the SMA being the most frequently treated vessel [6]. Across all cases, IVL demonstrated high technical success, with no reported complications such as vessel perforation, distal embolization, or thrombosis. Most procedures involved predilatation followed by IVL and stent implantation, approaches that mirror the technique applied in our case. The number of lithotripsy pulses varied widely (150–300), and both covered and uncovered stents were used, depending on vessel anatomy and operator preference [6, 12].

Our case adds to this evolving body of evidence by demonstrating the successful use of IVL as a preparatory step for stenting in the SMA. Importantly, given the patient’s oncologic status and the anticipated risk of postoperative mesenteric hypoperfusion, revascularization to restore luminal patency was essential. IVL facilitated successful low-pressure stent deployment. The use of a 5.5 × 60 mm Shockwave™ balloon and four cycles of intravascular lithotripsy in our case aligns with the range reported in previously published cases. Notably, this approach enabled the successful deployment of an 8 mm balloon-expandable stent, a diameter rarely achieved in the SMA due to its typically narrow caliber. Spath et al. reported stent implantation with a 7mm diameter following lesion preparation using an 8 × 60 mm IVL balloon. Remarkably, in our case, the deployment of an 8 mm diameter stent was achieved after preparation with a smaller 5.5 × 60 mm balloon, representing the largest reported discrepancy between IVL balloon and stent diameter in the current literature. This observation suggests that effective lesion preparation may be achievable even with smaller balloon diameters, underscoring the safety and efficacy of intravascular lithotripsy in addressing the biomechanical challenges posed by dense vascular calcification. Notably, IVL use in visceral arteries is off-label. Mesenteric case reports have not described IVL-related complications, but device-class data from other beds show low, but non-zero rates of dissection and rare perforation [5, 8]. Careful technique and ready bail-out options are therefore essential.

Off-label use warrants caution in specific high-risk situations: ulcerated soft plaque; aneurysmal, dissected, or fragile segments; short, rigid ostial lesions abutting the aorta; critical branches or poor collateralization; diffuse calcification with limited landing zones; and lack of surgical backup. Further, the risk profile specific to mesenteric circulation must be elaborated carefully before the intervention. Potential complications include dissection or perforation (including intramural hematoma), distal embolization with possible segmental bowel ischemia, vasospasm, and elastic recoil or residual stenosis requiring adjunctive therapy [2]. Given this risk profile, we selected a smaller IVL balloon for vessel preparation. Smaller diameters reduce wall stress and may lower the risk of previously mentioned complications. Compared with alternatives, cutting balloons can open resistant rings but are more invasive and carry a higher rupture risk at rigid ostia [4]. Atherectomy can debulk calcium but increases distal embolization and perforation risk and is often impractical for embolic protection in visceral branches [4]. Our case shows that even a relatively small IVL balloon can facilitate controlled plaque modification and enable full expansion of a large diameter stent, while potentially mitigating risks through a less traumatic vessel approach compared to alternative techniques.

This case supports the expanding utility of IVL beyond peripheral and coronary interventions, particularly in anatomically complex vascular territories such as the mesenteric circulation. Despite the encouraging results, the collective experience remains limited to a small number of patients. Further, this case report has several limitations. It describes a single patient and, by design, cannot establish causality or generalizability. Follow-up is limited to early imaging, so durability, restenosis risk, and clinical outcomes remain uncertain. Without a comparator (e.g., high-pressure balloon angioplasty), relative efficacy and safety cannot be inferred. Finally, extrapolation from coronary/peripheral data remains limited by differences in vessel size, ostial geometry, flow, collateralization, and the clinical impact of embolization in bowel-supplying arteries. Future prospective studies are warranted to assess long-term outcomes and better define selection criteria, especially in preoperative settings like ours.

## Conclusion

In conclusion, IVL represents a valuable adjunct for lesion preparation in the treatment of heavily calcified SMA stenoses, particularly when optimal stent expansion is required. This technique may be especially beneficial in the preoperative management of oncologic patients undergoing major abdominal surgery, where mesenteric perfusion must be safeguarded. Patients with prior failure of conventional high-pressure angioplasty or anticipated high rupture risk may also benefit from IVL. Further prospective studies are necessary to validate long-term outcomes.

## Data Availability

Not applicable.

## Abbreviations

SMA: Superior mesenteric artery
IVL: Intravascular lithotripsy
PTA: Percutaneous transluminal angioplasty
CTA: Computed tomography angiography
CMI: Chronic mesenteric ischemia

## References

[1] Hansen KJ, et al. Mesenteric artery disease in the elderly. J Vasc Surg. 2004;40(1):45–52. 15218461 10.1016/j.jvs.2004.03.022

[2] Huber TS, et al. Chronic mesenteric ischemia: clinical practice guidelines from the Society for Vascular Surgery. J Vasc Surg. 2021;73(1s):87s–115s. 33171195 10.1016/j.jvs.2020.10.029

[3] Lejay A, et al. Chronic mesenteric ischemia: 20 year experience of open surgical treatment. Eur J Vasc Endovasc Surg. 2015;49(5):587–92. 25728455 10.1016/j.ejvs.2015.01.004

[4] Terlouw LG, et al. European guidelines on chronic mesenteric ischaemia - joint United European Gastroenterology, European Association for Gastroenterology, Endoscopy and Nutrition, European Society of Gastrointestinal and Abdominal Radiology, Netherlands Association of Hepatogastroenterologists, Hellenic Society of Gastroenterology, Cardiovascular and Interventional Radiological Society of Europe, and Dutch Mesenteric Ischemia Study group clinical guidelines on the diagnosis and treatment of patients with chronic mesenteric ischaemia. United Eur Gastroenterol J. 2020;8(4):371–95. 10.1177/2050640620916681 PMC7226699 32297566

[5] Shammas NW, Mangalmurti S, Bernardo NL, et al. Intravascular Lithotripsy for Treatment of Severely Calcified Common Femoral Artery Disease: Results From the Disrupt PAD III Observational Study. J Endovasc Ther. 2024;0(0). 10.1177/15266028241255622. 10.1177/15266028241255622 38877777

[6] Spath P, et al. Use of shockwave intravascular lithotripsy in recanalization of calcified visceral and renal arteries: a case report and update of the literature. J Endovasc Ther. 2024;31(3):485–90. 36147019 10.1177/15266028221125157

[7] van Petersen AS, et al. Open or percutaneous revascularization for chronic splanchnic syndrome. J Vasc Surg. 2010;51(5):1309–16. 20304586 10.1016/j.jvs.2009.12.064

[8] Brodmann M, et al. Safety and performance of lithoplasty for treatment of calcified peripheral artery lesions. J Am Coll Cardiol. 2017;70(7):908–10. 28797363 10.1016/j.jacc.2017.06.022

[9] Beattie WS, et al. Survival after isolated post-operative troponin elevation. J Am Coll Cardiol. 2017;70(7):907–8. 28797362 10.1016/j.jacc.2017.06.023

[10] Brinton TJ, et al. Feasibility of shockwave coronary intravascular lithotripsy for the treatment of calcified coronary stenoses. Circulation. 2019;139(6):834–6. 30715944 10.1161/CIRCULATIONAHA.118.036531

[11] Adams G, et al. Intravascular lithotripsy for treatment of calcified lower extremity arterial stenosis: initial analysis of the Disrupt PAD III study. J Endovasc Ther. 2020;27(3):473–80. 32242768 10.1177/1526602820914598 PMC7288854

[12] Balboa Arregui O, et al. Use of shockwave intravascular lithotripsy for the treatment of symptomatic and severely calcified superior mesenteric artery stenosis. CVIR Endovasc. 2021;4(1):53. 34128127 10.1186/s42155-021-00243-5 PMC8203768

